# Sequential consumer choice as multi-cued retrieval

**DOI:** 10.1126/sciadv.abl9754

**Published:** 2022-02-25

**Authors:** Adam N. Hornsby, Bradley C. Love

**Affiliations:** 1 Dunnhumby, 184 Shepherds Bush Road, London W6 7NL, UK.; 2 Department of Experimental Psychology, University College London, London WC1H 0AP, UK.; 3 The Alan Turing Institute, London UK.

## Abstract

Whether adding songs to a playlist or groceries during an online shop, how do we decide what to choose next? We develop a model that predicts such open-ended, sequential choices using a process of cued retrieval from long-term memory. Using the past choice to cue subsequent retrievals, this model predicts the sequential purchases and response times of nearly 5 million grocery purchases made by more than 100,000 online shoppers. Products can be associated in different ways, such as by their episodic association or semantic overlap, and we find that consumers query multiple forms of associative knowledge when retrieving options. Attending to certain knowledge sources, as estimated by our model, predicts important retrieval errors, such as the propensity to forget or add unwanted products. Our results demonstrate how basic memory retrieval mechanisms shape choices in real-world, goal-directed tasks.

## Teaser

Five million sequential consumer choices are predicted by their similarity across multiple systems in memory.

## INTRODUCTION

Many studies of preferential choice have examined how people choose among a fixed menu of options (*1*–*3*). However, in real-world tasks such as online grocery shopping, the space of possible options is too large to be considered at once. Choices therefore depend on how options are retrieved from long-term memory (*4*–*7*). The task itself can provide the context to retrieve associated choice options. Here, we consider how a previous choice influences subsequent consumer choices in a goal-directed sequential decision task. Using computational models, we evaluate how different sources of knowledge influence choice by decomposing associative memory into its constituent components.

Once items are retrieved from memory, they may cue subsequent retrievals, leading to complex sequential dynamics such as semantic clustering. For example, when asked to name as many animals as possible, sequential retrievals tend to be semantically similar and faster when they are so (e.g., dog → cat) (*8*–*12*). This sequential cuing of memory means that retrievals tend to cluster over time. Sequential consumer choices may also semantically cluster if they depend on the same retrieval mechanisms; we test this hypothesis here.

Retrieval is said to depend on the strength of associations in memory, although “association” is somewhat nebulous given that items can relate in different ways. For example, choosing tea could trigger childhood memories of enjoying it with cake, as it did for Proust (*13*). Options that occur in the same episode could have a high probability of being retrieved; this was shown in the early experiments of memory (*14*) and has since become a core prediction in models of memory search (*15*).

Sequential choices could also be influenced by semantic similarity between items, such as their conceptual overlap. For example, while they may not be consumed in the same episode, purchasing chocolate could prompt the search for other chocolate bars because of their shared features. Semantic space models have been shown to predict sequential retrievals in fluency tasks (*11*, *16*) and options generated to open-ended questions (*7*). Online shoppers may similarly retrieve products that are nearby in conceptual space when making sequential choices, given that they are not constrained by the physical layout of products in stores.

An often-cited feature of semantic memory is that people are sensitive to hierarchical relations between items. For example, responses tend to be slower when judging the correctness of statements such as “apples are fruit” compared to “apples are produce” (*17*). One might therefore expect online shoppers to retrieve items that are nearby within a structured hierarchy, such as purchasing fruit then vegetables. This seems particularly likely during grocery shopping, given that stores tend to arrange products taxonomically to make them easier to locate (questions concerning whether hierarchical, semantic, and episodic knowledge are strictly separate systems from neurobiological or computational perspectives are orthogonal to our aims and claims).

**Fig. 1.**
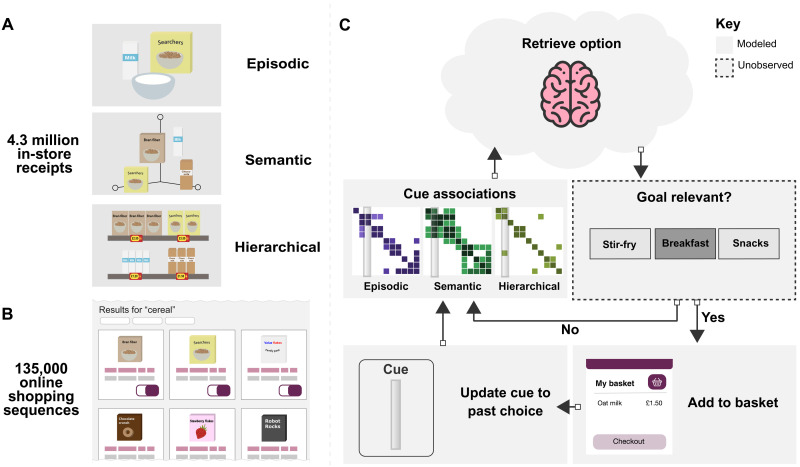
Deciding what to choose next when shopping for groceries online depends on cued retrieval from multiple knowledge sources. (**A**) We used 4.3 million unordered, in-store receipts to build representations of episodic, semantic, and hierarchical knowledge. (**B**) To model retrieval, we collected data from 135,000 shoppers as they sequentially searched for products on the website of one of the United Kingdom’s largest supermarket retailers. (**C**) Prior choices predict future ones, by virtue of their similarity according to different representational formats. Once an item is added to their basket, shoppers use this to cue matches from long-term memory. The stronger the match with this cue, the higher the probability an item will be retrieved (this may be attenuated by increased attention toward a particular representation). Retrieved items are checked against one’s internal goals. If the retrieval is goal relevant, then the shopper adds an appropriate item from the website and uses that item to cue associations. If not, then a new option is retrieved and checked for goal relevance until one is accepted. Similar heuristic strategies have been used in models of option generation for single choices (*18*, *19*). Once all goals are satisfied, the user checks out. Note that the goal-checking process is not modeled here.

We hypothesize that retrieval of options in sequential choice tasks depends on their similarity with the prior choice across different knowledge formats (visualized in Fig. 1C). We test this by developing associative representations of these knowledge sources [as in (*20*)] before evaluating whether sequential choices are better explained by one or a combination of these representations. We also hypothesize that individual differences may drive shoppers to attend to certain sources of knowledge more than others. For example, a shopper driven by episodic memories of breakfast might retrieve butter then bread, whereas those relying on hierarchical knowledge may retrieve butter with other dairy products, as they would in the supermarket. We operationalize these processes of associative retrieval and attention in a computational cognitive model and show that it can predict sequential consumer choices. After each retrieval, we suggest that consumers accept or reject possibilities according to their goals. For example, shoppers may consider whether retrieved options are suitable for breakfast. However, goals are not modeled or enumerated here, as we focus on the contribution of different knowledge systems during sequential option retrieval.

During online grocery shopping, consumers could feasibly search for products in any order. However, if options are retrieved according to their similarity with the prior choice, then purchases should be nonrandom and predicted by their sequential similarity. We test this using a new dataset of more than 5 million consumer choices. To foreshadow, results supported this hypothesis. Representations of episodic, semantic, and hierarchical knowledge explained unique variance when predicting sequential choices and their response times, supporting the idea that shoppers query multiple sources of long-term knowledge. Consumers retrieving options from episodic memory appeared less prone to subsequently forget products, whereas those attending to semantic knowledge were less likely to add items to their basket that they did not otherwise need. Thus, individuals may recruit these systems to different extents, which may affect their ability to complete the task effectively.

## RESULTS

We analyzed a dataset of sequential consumer purchases gathered from one of the United Kingdom’s largest supermarket retailers. The data contained 5,238,469 choices from 132,146 unique visitors across 42,837 unique products (more information in Materials and Methods).

Shoppers were required to search sequentially for groceries to add to their virtual basket (the website is depicted in fig. S1). On average, they made 39.64 choices [95% confidence interval (CI) = [39.49, 39.79]]. The landing page displayed a generic selection of “special offers” (e.g., discounted products), which was used relatively infrequently to purchase products (μ_offers_ = 1.55, 95% CI = [1.53, 1.58]). Shoppers tended to search for products using a search bar, which was located at the top of every page (μ_searches_ = 23.36, 95% CI = [23.26, 23.46]). They could also use a category drop-down by hovering the mouse over a hyperlink saying “groceries” at the top of every page. This menu required users to navigate to the lowest level of three subcategories before viewing products (e.g., cupboard → cereals → healthy cereals). It was used relatively infrequently (μ_category_ = 3.02, 95% CI = [2.98, 3.05]). After navigating with the search bar or the category drop-down, shoppers would be shown a list of associated products, where they could purchase products or click on products for more information. Before checkout, they could also add products from a personalized recommender system that suggested products that might have been forgotten before checkout (μ_forgotten_ = 0.322, 95% CI = [0.3199, 0.327]). Visitors removed an average of 3.23 products from their basket before checking out (95% CI = [3.20, 3.27]).

To explain sequential transitions between choices, we developed representations of episodic, semantic, and hierarchical knowledge. A representation of episodic memory was derived by calculating the probability of two products co-occurring in the same basket using a separate dataset of in-store transactions in which products appear unordered. With the same dataset, we trained a distributed representation of semantic memory using word2vec (*21*) [similar to the one developed in (*20*)]. A representation of hierarchical knowledge was calculated using the retailer’s five-level product taxonomy, which groups products from small subgroups (e.g., apples) to increasingly large departments (e.g., produce). Uniquely, this hierarchy describes a taxonomy of is-of relations, which we used to define a measure of similarity as opposed to distance in a continuous semantic space (see section S1.2 for more information).

### Past choices cue subsequent retrievals

**Fig. 2.**
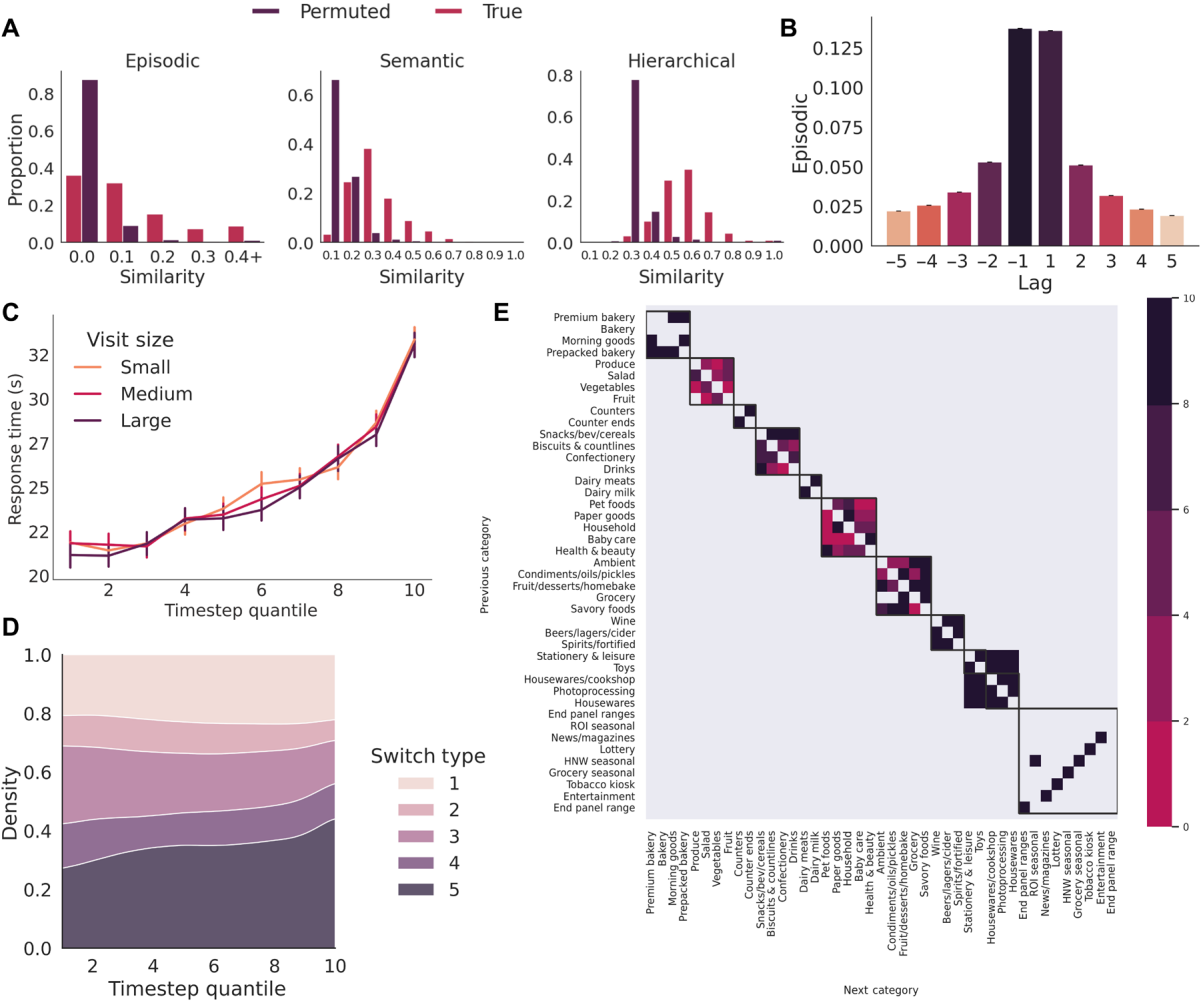
Consecutive purchases tend to be close episodic, semantic, and hierarchical relations. (**A**) Choices are predicted by their similarity with the prior choice across each representation. Histograms show that the similarity between consecutive purchases (averaged for each visit) was higher compared to when the order of purchases was randomly permuted (with 95% CIs). (**B**) Sequential retrieval is similar to a ripple in semantic memory. Mean episodic similarity (with 95% CIs) between the current product and those purchased recently is higher compared with products purchased later. (**C**) Visitors slowed as they approached the end of their shopping trip. Mean response times (with 95% CIs) as a function of timestep quantile (small, 10 to 30 items; medium, 31 to 49 items; large, 50+ items). (**D**) Consumers make more between-category transitions (i.e., taxonomy level five) toward the end of their visit. Stacked density plots denoting the proportion of switches according to each level of the taxonomy as a function of the relative timestep. (**E**) Transitions between product groups at the fourth level of the hierarchy clustered into intuitive higher-order groupings that appear similar to those in the product taxonomy, suggesting that the taxonomy closely resembles how shoppers represent products during sequential choice. The Lift-1 of each transition is depicted in purple, with values less than 0 shown in gray. Boxes represent clusters identified by the optimal spectral clustering solution (more information in sections S1.7 and S2.6). ROI, Republic of Ireland; HNW, Health & Wellness.

If shoppers cue retrievals from a given long-term store, then one would expect the similarity between consecutive purchases to be higher than when compared with random permutations, where the order of products has been randomly permuted within each shopping trip. For example, given that butter and bread are episodically linked (e.g., purchased in the same baskets), this should increase the probability of them being chosen consecutively. As shown in Fig. 2A, the average trip-wise similarity between consecutively purchased items was significantly higher for the true order of purchases compared to the permuted order for episodic (Median_true_ = 0.0756, IQR_true_ = 0.1465 and Median_permuted_ = 0.0154, IQR_permuted_ = 0.0193) (Mann-Whitney *U* = 2.95 × 10^11^, *P* < 0.0001, Common Language Effect (CLE) = 0.8312), semantic (Median_true_ = 0.2507, IQR_true_ = 0.1495 and Median_permuted_ = 0.0770, IQR_permuted_ = 0.0669) (Mann-Whitney *U* = 1.5 × 10^11^, *P* < 0.0001, CLE = 0.9141), and hierarchical representations (Median_true_ = 0.5169, IQR_true_ = 0.1433 and Median_permuted_ = 0.2620, IQR_permuted_ = 0.0504) (Mann-Whitney *U* = 1.07 × 10^11^, *P* < 0.0001, CLE = 0.9390). This suggests that choices were nonrandom and were cued by their similarity with the prior choice.

Sequential cued retrieval can be viewed as a ripple through memory, in that more recently retrieved items tend to be more similar (*11*). As shown in Fig. 2B, choices were most similar to the prior choice according to their episodic similarity (fig. S2 shows similar patterns for semantic and hierarchical knowledge). To confirm this, we calculated the average similarity of each choice for lags ranging from −10 to −1. Regressing lag onto the standardized average similarity for each user revealed a positive average relationship across each representation, indicating that more recent choices tend to be more similar (μ_episodic_ = 0.1357,95 % CI = [0.1331,0.1383], *Z*_sign_ = 56229.0, *P* < 0.0001; μ_semantic_ = 0.1837,95 % CI = [0.1816,0.1859], *Z*_sign_ = 59170.5, *P* < 0.0001; and μ_hierarchical_ = 0.2263,95 % CI = [0.2247,0.228], *Z*_sign_ = 61651.5, *P* < 0.0001).

The retrieval model described in Fig. 1 selects options according to the similarity with the previous option. As this process repeats, the chance that a high-similarity option has already been purchased increases, meaning that choices should become more dissimilar over time. Grouping choices into deciles based on their timestep (and thus adjusting for different trip sizes), we regressed each similarity measure onto timestep decile and included indicator variables of each transition type as confounding variables within each regression. Results showed that average similarity between sequential choices decreased over time across episodic (*b*_episodic_ = −0.058, 95 % CI [ −0.061, −0.055], *P* < 0.0001), semantic (*b*_semantic_ = −0.010, 95 % CI [0.013, −0.007], *P* < 0.0001), and hierarchical representations (*b*_hierarchy_ = −0.247, 95 % CI [ −0.250, −0.243], *P* < 0.0001) (full regression equations are in tables S3 to S5). The increase in hierarchical similarity over time is visualized in Fig. 2D.

One might correspondingly expect choices to become slower over time, as more dissimilar options are slower to retrieve. Regression analyses of interresponse intervals (IRIs) conformed to this expectation. First, the standardized coefficients for episodic (*b*_episodic_ = −0.137, 95% CI = [−0.138, −0.136], *P* < 0.0001), semantic (*b*_semantic_ = −0.086, 95% CI = [−0.087, −0.085], *P* < 0.0001), and hierarchical knowledge (*b*_hierarchy_ = −0.273, 95% CI = [−0.274, −0.271], *P* < 0.0001) were all negative predictors of IRI, indicating that more dissimilar options were slower to retrieve. Moreover, average IRIs appeared to slow over the duration of the trip (*b*_timestep_ = 0.130, 95 % CI [0.129,0.130], *P* < 0.0001). This slowdown is shown in Fig. 2C. Including variables representing the navigation method (e.g., keyword search) and the similarity across, each representation confirmed this slowdown as a general trend (full regression equation is in table S2), and this echoes similar patterns of slowing observed in category fluency tasks (*10*–*12*).

### Episodic, semantic, and hierarchical knowledge jointly explain sequential choice

We next evaluated whether consumers’ sequential choices were best explained by one or multiple sources of knowledge using the retrieval equation of a popular memory retrieval model, Search of Associative Memory (*22*). This equation formalizes how options may be retrieved on the basis of their similarity with the current cue (illustrated in Fig. 1C, with full equation in section S1.3). We evaluated its fit when including representations of episodic, semantic, and hierarchical knowledge. Results are presented as the mean improvement in the Bayesian information criterion (BIC) relative to a random model, for which the probability of each transition was equal across all remaining products (see section S1.3 for further details about the model fitting procedure).

As shown in Table 1, the best-fitting model contained multiple memory representations, even after penalizing for multiple parameters. This suggests that online grocery shoppers query multiple knowledge formats when deciding what to choose next. A model parameter recovery study revealed that each parameter could be recovered accurately, with correlations between actual and estimated parameters of >0.6 in all cases (reported in section S2.8). This indicates that parameter estimates were uniquely identifiable and could therefore be interpreted.

**Table 1.** The %BIC improvement over the random baseline and the mean attention weights (with 95% CIs) for each of the candidate models. Results show that including representations of multiple knowledge formats provides the best fit to the data (shown in bold).

	**ΔBIC (%)**	**Episodic**	**Semantic**	**Hierarchy**
Episodic	9.13	0.29 (0.001)		
Semantic	4.80		0.091 (0.001)	
Hierarchy	26.28			2.217 (0.016)
Episodic and semantic	12.30	0.258 (0.001)	0.068 (0.001)	
Semantic and hierarchy	29.10		0.055 (0.001)	2.105 (0.017)
Episodic and hierarchy	31.79	0.174 (0.001)		2.004 (0.017)
Multiple	**33.78**	0.160 (0.001)	0.044 (0.001)	1.939 (0.018)

Inspecting the average attention weights of the best-fitting model, one can gauge the relative importance of each representation. Hierarchical knowledge received the largest weight, followed by episodic, and then semantic knowledge. Further analyses (presented in section S2.6) revealed that transitions between product groups tended to overlap with superordinate classifications in the product taxonomy (clusters of transitions between product groups at the third taxonomic level are visualized in Fig. 2E). Together, this suggests that shoppers rely heavily on hierarchical knowledge about how products relate, which aligns closely with the taxonomy used to arrange products in stores.

We next evaluated whether response times were best explained by one or a combination of knowledge sources. A multiple linear regression was performed, predicting the IRIs between each choice, using each of the three similarity measures as predictors. We also included the number of products remaining to be purchased and used indicator variables representing each of the navigation methods (e.g., keyword search); these served as confounding variables (full model equation is in table S6). Model comparisons that penalized for more variables revealed that IRIs were best explained by this full model rather than one containing a subset of similarity measures (model comparisons are in table S7).

These results support our key claim that sequential choice in open-ended tasks depends on the retrieval of options from multiple sources of long-term memory. It is perhaps unexpected that episodic and semantic knowledge explain unique variance in consumer choices, given that the latter may derive from the former (*23*). However, episodic knowledge provides a more direct link between experiences than semantic knowledge, which may play a unique role during goal-directed choice. Most of all, shoppers appeared to depend on hierarchical knowledge about products, which emphasizes the influence of taxonomic organizations during navigation of large option spaces.

These model fits demonstrate the complementary role of different knowledge systems during everyday sequential choice tasks but should not be limited to such settings. For example, they should extend to more well-known experimental tasks, such as semantic fluency. To test this, we fit the same retrieval model to a separate dataset of sequential food retrievals collected in a controlled experiment [originally collected in (*24*) and shared via (*25*)]. In this task, 50 participants were given 3 min to retrieve as many food words as possible. Much like keyword searches, each retrieval was typed into a text box. For each word retrieved (e.g., “hamburger”), we found a corresponding product from the retailer, allowing us to measure the episodic, semantic, and hierarchical similarity between sequential retrievals as before (more details of the method and results can be found in section S3). After performing the same set of model comparisons, results showed that the best-fitting model contained all three representations, even after penalizing for the additional parameters. Moreover, much like shoppers, participants appeared to rely most on hierarchical knowledge when sequentially retrieving food items from memory. This suggests that these knowledge systems also influence sequential retrievals in controlled experimental tasks and that our model fits are representative of memory retrieval and not merely the design of the website.

### Relying on certain knowledge formats predicts retrieval errors

If shoppers rely on certain knowledge formats during retrieval, then this may increase their propensity to make certain errors such as forgetting or falsely retrieving products. Forgetting indicates the failure to retrieve a relevant item (i.e., a miss), whereas removing items indicates the failure to suppress irrelevant retrievals (i.e., a false alarm). Forgetting is often viewed as a failure of retrieval (*26*, *27*) and could simply result from “searching the wrong part of memory” [page 40 of (*28*)].

The retrieval model used here (*22*) assumes that items will be activated according to a process of spreading activation (*29*, *30*). When operating on an episodic representation, this would tend to chain together products found together in the same basket (e.g., purchasing a Thai pepper may cue coconut milk, bamboo shoots, and other complementary ingredients). Thus, we hypothesized that shoppers relying on episodic knowledge, as measured by the attention weights from the best-fitting retrieval model, would be less likely to forget products, as they would tend to coactivate items often combined in pursuit of a goal. Forgotten items were measured through the use of a recommender system, which displayed products that the shopper had purchased recently and frequently in prior visits before checkout.

When operating on a semantic network, a spreading activation process would tend to coactivate products that are substitutable or conceptually similar but not necessarily purchased together (e.g., purchasing a Thai pepper may coactivate other forms of pepper). We therefore hypothesized that shoppers relying more on semantic knowledge would be more prone to remove products from their basket, indicating that they did not actually need them. This shares a kindred spirit with theories of confabulation in memory retrieval (*31*, *32*), where high semantic similarity between studied items causes related items to be erroneously retrieved.

### Forgetting products

**Fig. 3.**
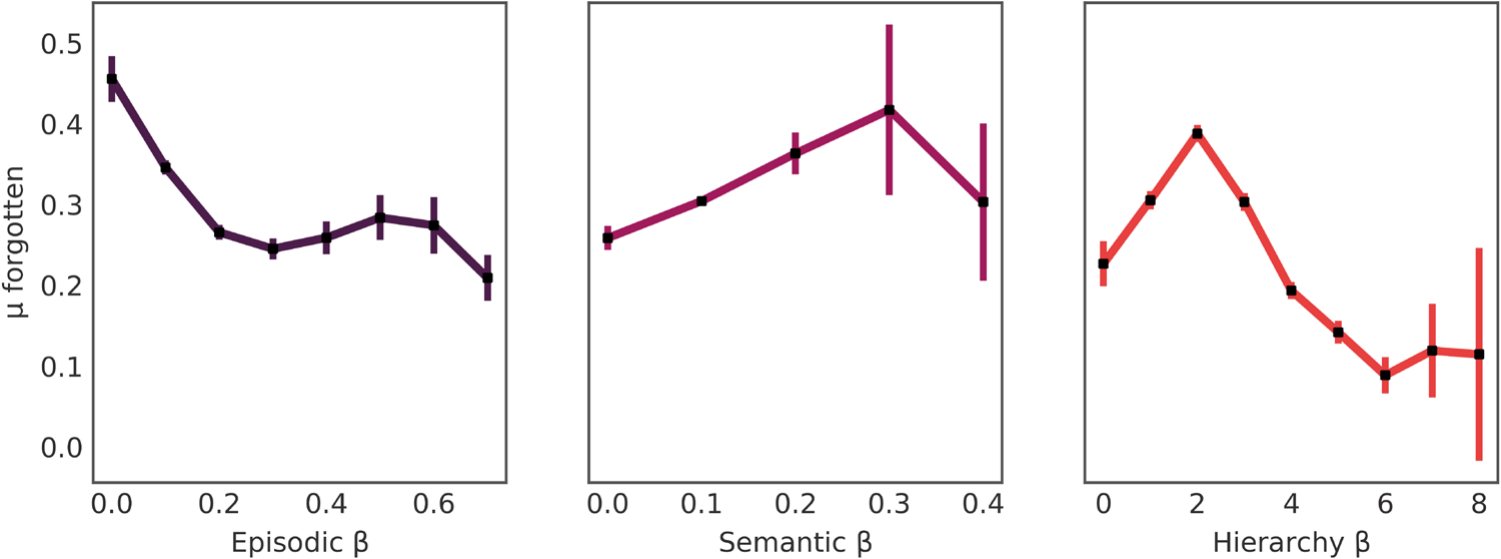
Mean number of forgotten items (with 95% CIs) for each model attention weight (β). Results show that relying on episodic or hierarchical knowledge predicted fewer forgotten items, whereas attending to semantic knowledge predicted more forgotten items, as measured by the use of a recommender system displayed before checkout.

As shown in Fig. 3, results supported the prediction that shoppers with increased episodic retrieval forgot fewer items on average (*r_s_* = −0.0811,95 % CI [ −0.0868, −0.0754], *P* < 0.0001). In addition, attending to hierarchical knowledge predicted fewer forgotten items (*r_s_* = −0.0088,95 % CI [ −0.0145, −0.0031], *P* ≤ 0.003). In contrast, the more shoppers attended to semantic knowledge, the more likely they were to forget items (*r_s_* = 0.0152,95 % CI [0.0095,0.0209], *P* = 0.0001).

To gauge their relative usefulness, we scaled each attention weight and entered them into a multiple linear regression, regressing onto the number of forgotten items. We also included the number of choices and the proportion of choices made using each search context as confounding variables. The regression was significant (*F*_8,117328_ = 178.7, *P* < 0.0001, *R*^2^ = 0.012). Higher attendance to episodic knowledge (*b*_episodic_ = −0.053, 95% CI = [−0.059, −0.048], *P* < 0.0001) or hierarchical knowledge (*b*_hierarchy_ = −0.041, 95% CI = [−0.046, −0.035], *P* < 0.0001) negatively predicted forgetting, whereas attending more closely to semantic knowledge (*b*_semantic_ = 0.015, 95% CI = [0.009, 0.021], *P* ≤0.0001) positively predicted forgetting (full regression equation is reported in table S9).

Relying on episodic knowledge not only generates more complementary retrievals but also could indicate greater experience with products and their relationships. Both could explain the general trend of forgetting fewer items as one increases attendance to episodic knowledge. Moreover, the formalization of episodic knowledge predicts that shoppers will transition to products that frequently co-occur with the past choice. Thus, another possibility is that the recommended products were less relevant to those that used episodic knowledge to guide their search because they had been purchased by that shopper with a high frequency in the past. More work, perhaps through a cognitive battery, is required to understand the relationship between retrieval from different knowledge systems and forgetfulness.

### Removing products

We next examined whether shoppers attending to certain knowledge sources removed more items from their basket. In line with our predictions, shoppers who attended more closely to the semantic similarity between items removed more items from the basket (*r_s_* = 0.0992,95 % CI [0.0935,0.1049], *P* < 0.0001). Conversely, those attending to episodic knowledge (*r_s_* = −0.014,95 % CI [ −0.0197, −0.0083], *P* < 0.0001) or hierarchical knowledge (*r_s_* = −0.084,95 % CI [ −0.0897, −0.0783], *P* < 0.0001) removed fewer items on average.

As before, we regressed each of these descriptors onto the number of items removed from each basket. We also included the number of choices and the proportion of choices made using each search context as confounding variables. The regression was significant (*F*_8,117328_ = 2202.0, *P* < 0.0001, *R*^2^ = 0.131). Increased attention to semantic similarity was shown to positively predict the number of items removed (*b*_semantic_ = 0.051,95 % CI = [0.012,0.089], *P* < 0.0001). Conversely, higher attendance to episodic knowledge (*b*_episodic_ = −0.359,95 % CI = [ −0.398, −0.319], *P* < 0.0001) and hierarchical knowledge (*b*_hierarchy_ = −0.870,95 % CI = [ −0.909, −0.831], *P* < 0.0001) predicted fewer removed items (full regression equation is in table S10).

Relying on semantic knowledge generates more substitutable retrievals, which could explain why these shoppers tended to add products to their basket that they do not otherwise need. Further analyses (presented in section S2.10.1) revealed that the removed items tended to have above-average similarity with the chosen options across each knowledge representation, which is supportive of this idea. Another possibility is that shoppers attending to episodic knowledge were more experienced with products and their associations and thus were less prone to mistakes. We leave these possibilities for future analyses.

These results provide further support for the main claim that shoppers query multiple knowledge formats when deciding what to choose next and that individuals may differ in the extent to which they rely on these systems. Future experimental work may wish to test these findings using explicit measures and thus causally evaluate the precise relationship between attention to representations and errors. Such studies would complement our claim that knowledge formats may be recruited by individuals to different extents.

## DISCUSSION

In open-ended choice tasks such as grocery shopping, how do we decide what to choose next? Many factors could influence what is chosen, but we propose that much depends on the similarity with the preceding choice across multiple knowledge formats. This view makes a number of predictions that we confirmed. First, choices and their response times were predicted by their similarity with the last choice, suggesting that choices cue the retrieval of subsequent options. Second, this behavior was best explained by a mixture of episodic, semantic, and hierarchical knowledge, suggesting that consumers reason about associations between products in different ways by querying different sources of knowledge. Third, how prone consumers were to different types of memory errors was predicted by their reliance on different types of memory, as assessed by model fits.

As our model describes, retrieved options may be cued by prior choices. This likely explains why the sequential choices of online grocery shoppers clustered over time, as they do in fluency tasks (*8*, *10*–*12*, *33*). We build upon past research (*4*–*7*, *34*) by showing how memory retrieval mechanisms influence the generation of options in sequential decision-making tasks. These results would not be predicted by many classical models of preferential choice, which consider option retrieval to be out of scope (*1*–*3*). Our results demonstrate how choice options can be dynamically constructed in the moment depending on the context supplied by the previous choice. Future work could explore the influence of other past retrievals, which have been shown to influence list recall [for a review, see (*15*)].

Choices may follow different trajectories depending on which sources of knowledge are queried. Overall, choices and their response times were best explained by the sequential similarity across episodic, semantic, and hierarchical representations. In addition, individual differences in the extent to which each representation was recruited predicted how many products would be forgotten or removed. This would not be predicted by many existing models of semantic memory retrieval (*11*, *12*) and option generation (*6*, *7*), which rely on a single measure of association. Associative knowledge likely takes several forms [e.g., see (*35*)], which is consistent with our modeling approach and results. Future experimental work may wish to explore the role of different associations in memory retrieval tasks and whether such systems are cognitively or neurally distinct.

Although we focused on sequential retrieval of choice options, determining whether an option is goal relevant could also be key to choice (see Fig. 1C). While modeling goals was out of the scope of this study, we hope that studying the interaction between goals and retrievals will be addressed in future work. A person’s subjective preferences may also affect which retrieved options are chosen (*6*, *36*, *37*). Choice itself can affect preferences (*37*), which, in turn, may affect memory retrieval. For example, new episodic memories could be formed after purchasing a preferred pairing of balsamic vinegar and bitter salad. One exciting direction for future research is to consider how different shopping experiences for individuals lead to different memory representations, which, in turn, affect future purchasing decisions.

One possible confounder is that sequential choices were biased by the design of the website. For example, adding different brands of cola from the same page could cause retrievals to appear more hierarchical, as they belong to the same subcategory. To test this, we reran all analyses on a filtered dataset of product transitions that occurred through the use of the search bar (detailed in section S2.11). All results were consistent with those reported here, which is reassuring given that these transitions were perhaps best representative of memory-based search. In addition, the model that best explained sequential grocery choices also provided the best fit to sequential retrievals of foods, which were observed in a controlled laboratory task (detailed in section S3). Thus, while design features may help shoppers retrieve certain brands (e.g., brands of cola), shoppers still seem to depend on cued retrieval from multiple knowledge formats to determine what they will look for next.

Analyses of large field data such as these complement findings from the laboratory, allowing theories of memory and cognition to be evaluated at an unprecedented scale with high ecological validity. In this case, we have shown that the sequential purchases of grocery shoppers are well explained by a model of memory retrieval that was originally developed to explain behavior in laboratory tasks (*11*, *22*). A large driver of this model’s success in this task is that it makes use of three relevant embedding spaces that relate to knowledge systems proposed in studies of memory (*38*). We hope that these findings stimulate further work in the laboratory, where one typically has a higher degree of control for assessing questions of cause and effect. For example, an additional explanation for choices becoming slower and more dissimilar over time is that retrieved options are increasingly rejected as they become less goal relevant (e.g., goals become increasingly satisfied). Future laboratory studies could assess this claim by asking participants to choose options in the presence of more or fewer goals. Others could inquire about the content of people’s goals and examine how they interact with choices over time.

Online shoppers may be more or less responsive to certain recommendations depending on their navigational strategy. Results showed that shoppers relying on episodic memory were less likely to purchase products from a recommender system that reminded shoppers of previous purchases before checkout. This may be of practical significance to marketers designing personalized recommender systems, who could adapt recommendations to suit the retrieval strategies of shoppers as estimated by our cognitive model. For example, shoppers relying on hierarchical knowledge could benefit from recommendations promoting episodically related products (e.g., “goes well with...”), whereas those relying on episodic knowledge could benefit from seeing semantically similar products (e.g., “people also viewed...”). Such insights would complement traditional machine learning systems, which do not typically consider variations in human cognition (*39*).

Our approach may make it possible to use shopping behavior to detect cognitive impairments. Longitudinal studies link performance in retrieval tasks to memory decline in preclinical Alzheimer’s disease populations (*40*). While many people shop, relatively few people participate in such clinical tests until they experience serious memory impairment, thereby foregoing the advantages of an early diagnosis (*41*). Although more work would be needed to establish efficacy and suitable ethical guidelines, model fits (e.g., changes in attendance to episodic memory cues) may, in the future, predict the onset of cognitive impairment. Such a system operating at scale with informed consent could improve outcomes for individuals and society.

To conclude, our findings suggest that grocery shoppers use their previous choice to query associations across multiple knowledge systems when determining their next purchase. Depending on which sources of knowledge are queried, shoppers may choose products in different orders or exhibit an increased propensity to forget. Working with models and memory formats originally developed in laboratory settings, we were able to verify and extend these ideas in a real-world setting. In doing so, we strengthen the case for the complementary nature of laboratory and large-scale, real-world studies (*20*, *42*) with linkages enhanced through common modeling approaches.

## MATERIALS AND METHODS

### Clickstream data

Data capturing a sequence of clicks during a given shopping session are known as clickstream data. In this study, we used clickstream data collected by a large British retailer between 1 January 2015 and 31 March 2016. We used a random sample of visits resulting in a checkout during that period, each from a different customer, and only kept observations where a product was added to a shopper’s basket (more information in section S1.1). By shopping online, all customers were required to participate in the loyalty scheme of the retailer and therefore consented to having their data used for research. To preserve user privacy, we removed all customer identifiers from the data and kept only a cryptographic hash of each visit ID. All analyses were in compliance with University College London’s code of ethics.

### In-store data

To prevent information leaking into our analysis of online shopping behavior, we used a distinct dataset of in-store shopping behavior to develop knowledge representations. In-store grocery receipts are unordered, making it particularly useful for this study. The final dataset contained purchase information from 4,336,917 distinct baskets. We followed the same procedure of encryption as with the clickstream data to preserve the privacy of customers.

### Forgotten items

Forgotten items were flagged through the use of a personalized recommender system, which prompted users about items that they may have forgotten at the end of their visit, before they checked out. The exact products shown to each customer were determined according to the recency and frequency of purchase in previous shops (online or in-store), deduplicated against products that had been purchased in the present visit. This page was displayed to users before payment.

### Removed items

Shoppers could also remove products from their basket at any time during the shop. The total number of removed items was counted for each user. Further details about the method can be found in section S1.

## Supplementary Materials

### This PDF file includes:

Sections S1 to S3 Figs. S1 and S2 Tables S1 to S21 References
