# Supplementary material for: Sequential consumer choice as multi-cued retrieval
Source: Sci Adv. 2022 Feb 25;8(8):eabl9754. doi: 10.1126/sciadv.abl9754 (PMC8880769; doi:10.1126/sciadv.abl9754)
Supplement: Supplementary file 1 — Sections S1 to S3 Figs. S1 and S2 Tables S1 to S21 References [file sciadv.abl9754_sm.pdf]

Science Advances
AAAS

Supplementary Materials for

Sequential consumer choice as multi-cued retrieval

Adam N. Hornsby* and Bradley C. Love

*Corresponding author. Email: adam.hornsby.10@ucl.ac.uk

Published 25 February 2022, *Sci. Adv.* **8**, eabl9754 (2022)
DOI: 10.1126/sciadv.abl9754

This PDF file includes:

Sections S1 to S3
Figs. S1 and S2
Tables S1 to S21
References

1 Method

1.1 Data

1.1.1 Clickstream data

A sample of the total visits was used at the request of the retailer. Visits were then filtered such that only those resulting in a purchase were kept. We then filtered the observations to instances where the visitor added an item to their basket, rather than — for example — viewed without adding. If products were later removed from a basket, they were not removed from the dataset, as we wanted the order to reflect the original retrieval process of the shopper. Unlike standard fluency tasks, repeated retrievals cannot be considered erroneous and thus were included in the analyses. Each visit was from a unique visitor to ensure that visits were independent.

Note that 12,520 (0.23%) basket adds in the clickstream data were for products sold exclusively online. Pairwise similarities were therefore not computable for these observations. For any analyses that included pairwise similarities, these non-computable pairwise similarities were dropped. This left 5,238,469 basket adds from 132,146 unique visitors.

Visitors could navigate between products using different features of the website, which are visualised in Figure S1. Most choices were located using the search bar located at the top of the page, either immediately after its use (i.e., *search arrival*) or after a previous purchase in this context (i.e., *search stay*). Less often, visitors located products using the category drop-down (i.e., *category arrival*) or after a prior purchase in this context (i.e., *category stay*). Products could also be located using a generic special offers page, which was located on the landing page and could also be located using the ribbon at the top of the page. Finally, before checkout, visitors were suggested products that they may have forgotten.

1.1.2 In-store data

We used a sample of baskets purchased in-store during the same dates as the online dataset. Baskets were filtered so that they contained at least one of the products purchased in the clickstream dataset and contained a minimum of 5 products overall (a similar strategy was used by (20) to ensure that there was enough co-occurrence within each basket to learn reasonable representations of similarity).

1.2 Representations of associative knowledge

As explained in the introduction, we evaluated the relative contributions of three knowledge sources during sequential retrieval.

1.2.1 Episodic knowledge

The long-term episodic retrieval structure used in the Search of Associative Memory (SAM) model (22) associates items more strongly to the extent that they co-occur during encoding. Whilst we cannot know the exact context in which consumers encoded products, we assume that products purchased together more frequently in the same basket must be stronger episodic associates. Episodic associations are therefore represented using the pairwise co-occurrence between products observed in the in-store dataset.

Episodic similarities $S(a, b)$ were determined using the probability of co-occurrence between products a and b, which is given by:

$$S(a, b) = \frac{frq(a, b)}{frq(a)}$$

Where $frq(a, b)$ is the total number of number of times that product a co-occurred with b in the same basket across the dataset.

1.2.2 Semantic knowledge

In addition to episodic memory, shoppers likely also rely on semantic memory to guide their retrievals. A common feature of modern semantic memory models is that they represent knowledge within a connected representational space, allowing people to generalise their knowledge to observations they haven't directly experienced (43). Unlike episodic co-occurrence, two items that have never co-occurred together may still be considered semantically similar, so long as they co-occur in similar contexts. We followed recent research (20) by training a 200-dimensional distributed semantic model using the in-store data.

For this project, we chose to learn 200-dimensional vector representations for products with *word2vec* (21). This is because *word2vec* tends to scale better when trained on large datasets, because it can be trained stochastically. Rather than encoding words (e.g., as they might appear in the product descriptions), *word2vec* was trained to represent supermarket product codes, as they might appear in till receipts. Concretely, there is a different product code for each distinct product in the supermarket. Small variations in that product (i.e. different sizes of the same t shirt) are not given separate codes however. Each product code was thus represented as a one-hot encoded vector before being embedded, which resulted in a $42,837 \times 200$ matrix. During training, the model learns to associate product codes that often co-occur in baskets, which is analogous (but not the same) to how word vector models learn similarity between word tokens that often co-occur in sentences.

Associations S between any two vectors v_1 and v_2 was calculated using the cosine similarity $cos(v_1, v_2)$:

$$S(v_1, v_2) = cos(v_1, v_2) = \frac{v_1 \cdot v_2}{||v_1|| \cdot ||v_2||}$$

Conventionally, *word2vec* is trained using one of two network architectures. In this case, we used Continuous Bag of Words (CBOW) with negative sampling. CBOW assumes that items within baskets are un-ordered (i.e., known in natural language processing as the "bag of words" assumption). This assumption is true of in-store supermarket data, where product codes within baskets are unordered once they reach the database. Using CBOW, the training objective O is to maximise the likelihood of a target item i_j given a window of c surrounding context items:

$$O = \frac{1}{T}\sum_{j=1}^{T} logp(i_j|i_{j-c}, ..., i_{j+c})$$

Where T is the corpus size (e.g., number of baskets) and $p(i_j|i_{j-c}, ..., i_{j+c})$ is the probability of a target item i_j given average or summation of a set of context words $(i_{j-c}, ..., i_{j+c})$. In this case, word2vec used a context window size of 15.

Whilst this probability *could* be determined using a softmax, it is not practical when called over large vocabulary sizes. Negative sampling mitigates this by randomly sampling a set of k "negative" items (in our case, 20) that did not appear in the present basket. The model then learns from these negative samples by treating them as false labels in multiple binary classification tasks, evaluated using a logistic loss and then updated using gradient descent. In this project, this training process was repeated for 15 epochs.

We determined these aforementioned hyperparameters by monitoring the loss on the training set and visually inspecting the nearest products in terms of cosine similarity for a set of randomly-chosen products. During model fits, negative cosine similarities were clipped to 0.

1.2.3 Structured hierarchical knowledge

A strict organisation of products is imposed on consumers by way of a product taxonomy, which groups products from small subgroups (e.g., apples) to large departments (e.g., produce). Amongst other things, this taxonomy determines the proximity of products on the shelves and aisles of a supermarket store. The product taxonomy used here contained five levels. Each product had a unique taxonomisation.

If products a and b shared the same low-level category within the taxonomy, then they were said to be perfectly associated $S(a, b) = 1$. Products with entirely different taxonomic classifications had $S(a, b) = 0.2$. The remainder increased in increments of 0.2.

1.3 Retrieval model

Broadly, our retrieval model is based on the retrieval equation from Search of Associative Memory (SAM) (22). It assumes that retrieval and thus the decision of what to choose next is achieved by querying associative structures in memory with a memory probe. We follow previous models of semantic fluency by using the most recently chosen option O_i to probe associative memory structures (11, 12). Whilst other possibilities exist — such as a decaying influence of

all previous retrievals — we focus on the role of the prior choice in order to simplify analyses (for a review of other approaches, see 15). The retrieval strength of the subsequently chosen option O_{i+1} is given by the product of the M associations between the present choice and itself, $S(O_i, O_{i+1})_j$. For example, in the full model, we used episodic, semantic and hierarchy-based associations between products, meaning that $M = 3$. This is then divided by the sum of that same function applied to all of the N options that remain to be added for that trip. This then gives rise to an overall probability of retrieval for each choice:

$$P(O_{i+1}|S_1, S_2, ..., S_j, O_i) = \frac{\prod_{j=1}^{M} S(O_i, O_{i+1})_j^{\beta_j}}{\sum_{k=1}^{N} \prod_{j=1}^{M} S(O_i, O_k)_j^{\beta_j}} \tag{1}$$

We compared the inclusion of episodic, semantic and hierarchy-based associations. β values represent attention weights for each of these knowledge representations and were estimated as free parameters for each visit.

Each model was compared with a random baseline model, which predicted an equal probability of $\frac{1}{N}$ for every transition using a single representation. Thus, each of the products remaining to be purchased by each visitor is assumed to have an equal probability of being chosen at each timestep according to the baseline model.

1.3.1 Fit procedure

Each measure of association j was raised to its own respective attention weight β_j; these were treated as free parameters and fit to individual visitors using maximum likelihood estimation (attention weights were forced to have a lower bound of 0, in order to prevent individual retrieval probabilities from exceeding 1). These free parameters were solved separately for each visit using the SLSQP solver within SciPy.

1.3.2 Model input

Models were fit to the retrieval sequences in the clickstream data. In addition to non-computable similarities, observations were dropped from the clickstream data if they occurred during or after the use of a recommender system, which prompted users about items they may have forgotten before checkout. Finally, to ensure that parameter estimates were robust, visits were dropped if they contained fewer than 10 items. This left 117,337 distinct visits.

Because of the probabilistic and multiplicative nature of the model, negative or zero similarities were replaced with a very small but positive number $1e - 7$.

1.4 Permutation tests

To assess whether subsequent retrievals were more related than would be expected at random, we performed a permutation test. Each product within the clickstream data was encoded with each of the three representations described above. We then calculated the per-visit mean similarity between consecutively added products. These were compared to the per-visit mean similarities determined by 100 random permutations of the product order, permuted within each visit. Thus, for significance tests reported; $N_{true} = 132,146$ and $N_{permuted} = 13,214,600$.

1.5 Response times

Response times (RTs) were compared for transitions of varying distances. These were capped at 60 seconds to minimise the leverage of outliers.

In the multiple linear regression comparing each similarity measure, RTs were monotonically transformed using a log function, $RT_{log} = \ln(RT + 1)$, due to positive skewness.

1.6 Trajectory analyses

Correlational analyses were conducted to assess how behaviour changed over time. Because visits contained differing numbers of products, basket adds within each visit were binned into equally-sized deciles based on their proximity to checkout. Subsequently, 13,901 (10.52%) visits were dropped because they purchased fewer than 10 products, leaving 5,174,018 choices.

1.7 Transition clustering

Spectral clustering was used to determine the extent to which transitions between categories within the taxonomy were clustered and thus could be predicted based on features of the current choice.

For each level of the product taxonomy, we calculated a transition matrix, counting the number of one-step transitions from each category i to every other category j (e.g., apples $\rightarrow$ pears). Because product sales tend to be Pareto distributed (e.g., products such as milk and bananas are considerably more popular), we found that the odds of transitioning to more popular products were disproportionately skewed. To adjust for this, we used the Lift association score, which is used in Market Basket Analysis (44). Lift is defined as

$$Lift(i,j) = \frac{Support(i,j)}{Support(i)\ Support(j)},$$

where

$$Support(i,j) = \frac{Frq(i,j)}{N}$$

is the probability of the i to j transition out the N transitions observed.

The denominator in the lift calculation $Support(i)\ Support(j)$ therefore describes an expected probability of a particular transition being made, given the overall popularity of the two respective categories. Thus, lift values above 1 for a given transition suggest that shoppers transition between these categories more than one would *expect*. Because only higher-than-expected transitions were of interest for these analyses, we subtracted 1 from the lift matrix and set the lower bound to 0:

$$Lift(i,j) = \begin{cases} 0 & Lift(i,j) - 1 \leq 0 \\ Lift(i,j) - 1 & \text{otherwise} \end{cases} \tag{2}$$

This lift association matrix of the transitions can be thought of as the adjacency matrix of a directed graph. In this context, spectral clustering is a natural candidate for our problem, as it is commonly used in network science to identify k clusters (or *communities*) within a graph. The algorithm works by first calculating the normalized Laplacian of the graph's adjacency matrix and then applying a standard clustering algorithm (such as k-means) to the relevant eigenvectors of that new space. Similar approaches have been used in reinforcement learning to abstract high-level sub-goals from transition matrices and in recent neuroscientific literature to understand how the hippocampus represents actions to facilitate spatial navigation (45). These problems are analagous to the one faced by shoppers here, hinting that there may be a deeper connection between these clusters and the planning processes of shoppers; we leave this possibility open for future work.

1.8 Predictive modelling

We explored whether the attention weights (β) from the best-fitting retrieval model would predict the number of forgotten or removed items.

Attention weights (β) were taken from the best-fitting retrieval model, and reflect the extent to which each visitor recruited each of the three representations to guide choice. Importantly, these weights were estimated using behaviour prior to the use of the recommender system. Outlying attention weights (three standard deviations above the mean) were clipped for this analysis for numerical stability.

2 Additional analyses of clickstream data

2.1 Correlations between representations

A key claim in this article is that people flexibly recruit multiple representations when deciding what to retrieve next. For this to be the case, each similarity measure would need to be related but not perfectly correlated. Looking at the sequential choices observed in the clickstream data, Spearman correlations revealed a moderate to strong relationship between each measure (coefficients shown in Table S1. All were significant $p < .001$). Thus whilst these similarity measures are directionally similar, each likely captures unique information relevant to retrieval.

2.2 Similarity ripples

The results shown in Figure S2 show that option retrievals can also be viewed as a ripple through semantic and hierarchical knowledge, in addition to episodic memory.

2.3 Timestep and IRI regression

To evaluate whether responses slowed down over time, we regressed timestep onto IRI using a linear mixed-effects regression. We included the visit identifier as a random effect and included dummy coded representations of each navigation method as confounding variables. This was to confirm that any slow-down was not simply a result of the website's design. We also included each measure of similarity, to ensure that — holding the similarity between transitions constant — choices because slower over time. The multiple linear regression converged ($ll = -5979127.00$). As shown in Table S2, the partial regression coefficient for timestep was positive, indicating that choices became slower over time, irrespective of how choices were navigated and the similarity between choices in memory.

2.4 Similarity and timestep regression

To evaluate whether choices became less similar over time, we regressed each similarity measure onto timestep using linear mixed-effects regressions. In each regression we included the visit identifier as a random intercept and included dummy coded representations of each navigation method as confounding variables. The regression predicting timestep using episodic similarity converged ($ll = -12751137.48$) and the coefficients are shown in Table S3. The regression predicting timestep using semantic similarity also converged ($ll = -12751890.27$) and the full equation is shown in Table S4. The regression predicting timestep using hierarchical similarity also converged ($ll = -12743140.39$) and the full equation is shown in Table S5. Each regression revealed a negative relationship between similarity and timestep, indicating that — irrespective of the navigation method — sequential choices became more dissimilar over time.

2.5 Representation and IRI regression

A mixed-effects multiple linear regression was performed, regressing each similarity measure onto the IRIs between each choice. We included a random intercept for each visit. We also — as above — included dummy coded representations for each navigation method and counts of the number of choices remaining as confounding variables.

The coefficients are presented in Table S6. They reveal that — taking account of different navigation contexts — retrieval from hierarchical knowledge uniquely explains the most variance in response times of the knowledge types, followed by episodic knowledge and then semantic knowledge.

2.5.1 Model comparison

We performed feature selection for the inter-response interval (IRI) mixed-effects linear regressions, to confirm that response times were best explained by multiple knowledge systems. Similarity measures were removed using stepwise-elimination. All models contained the confounding variables described above. We compare models based on their Akaike Information Criterion (AIC) and Bayesian Information Criterion (BIC).

Table S7 indicates that each measure of similarity contributed significant explanatory power to the model, with full model having the lowest AIC and BIC overall. This indicates that choice IRIs are best explained by a combination of knowledge sources.

2.6 Transitions between categories reveal hierarchical knowledge

To gain further insight about the structure of shopper's hierarchical knowledge, we evaluated whether sequential transitions clustered into meaningful groups. In particular, we clustered transitions between product groups at each level of the product taxonomy. Transitions between categories appeared clustered — at least to some extent — across all levels of the product taxonomy. All of the best-fitting clustering solutions exhibited a positive silhouette score (Level 3: $Silhouette = .503$ $N_{clusters} = 23$, Level 4: $Silhouette = .614$ $N_{clusters} = 11$, Level 5: $Silhouette = .33$ $N_{clusters} = 4$). For example, transitions between categories defined at the fourth level of the product taxonomy, depicted in Figure 2e in the main manuscript, appeared highly clustered. Moreover, the clusters revealed intuitive groupings that often overlapped with super-ordinate classifications in the product taxonomy (e.g., clustering separate beers, wines and spirits categories). One possibility is that these clusters emerge because shoppers transition between them with a clear plan in mind, which is executed across multiple transitions. However, a simpler explanation could be that these clusters emerge as a consequence of cued-retrieval from a hierarchical associative knowledge structure.

Although shoppers could feasibly transition between any pair of products for a comparable physical cost, it's intriguing that they instead prefer to adhere closely to the product taxonomy. One possibility is that shoppers use a mental model of a physical store layout to guide their search online, indicating a close correspondence between spatial and non-spatial navigation. Another related possibility is that this product taxonomy has been developed to closely resemble the knowledge structures of consumers.

2.7 Correlation between attention weights

Table S8 shows the correlations between each of the learned attention weights across all visits (all were significant $p < .0001$). Despite reasonably high correlations between representations, it is perhaps reassuring that correlations between attention weights do not far exceed $|0.28|$.

2.8 Retrieval model parameter recovery

To ensure that each knowledge representation was identifiable, we performed a parameter recovery study. We took a random 10% sample of visits and attempted to recover the attention weights that had been learnt during estimation of the multiple parameter model on the clickstream data. For each set of attention weights, we generated 100 synthetic purchase sequences, by selecting a random product as the first choice and then sampling subsequent choices according to the retrieval model. Choices were selected from the product universe observed in the clickstream dataset and product repetitions were permitted. Each generated trip contained 40 products, which is equal to the average purchase length observed in the true data.

Results showed that each parameter could be recovered accurately. In particular, Spearman correlations between estimated and actual β weights were high across episodic ($r_s = 0.6829, p <= .0001$), semantic ($r_s = 0.6034, p <= .0001$) and hierarchical knowledge ($r_s = 0.9883, p <= .0001$). That each system can be uniquely identified supports our interpretations of these knowledge sources as distinct cognitive processes.

2.9 Forgotten items regression

As reported in the main text, we regressed attention weights from the best fitting model onto the number of forgotten items using multiple linear regression. We also included the total number of choices and the proportion of each transition as confounding variables. The full model equation is shown in Table S9. These results show that the model attention weights explained unique variance when predicting the number of forgotten items. This suggests that — irrespective of how one navigates the website — the proximity between sequential choices in memory predicts one's propensity to forget products.

2.10 Removed items regression

Similarly, we regressed attention weights from the best fitting model onto the number of products removed from each basket. We also included the total number of choices and the proportion of each transition as confounding variables. The full model equation is shown in Table S10. These results show that the model attention weights explained unique variance when predicting the number of products removed. Similarly, this suggests that — irrespective of how one navigates the website — sequentially purchasing products that are close semantic relations may increase one's propensity to add items to their basket that they don't otherwise need.

2.10.1 Similarity between removed and purchased products

An alternative explanation for removing products could be that they were random products added accidentally or that they ended up not complementing the rest of a shopper's basket and thus being very distinct. To evaluate these possibilities, we evaluated the similarity between removed products with those purchased in each visit. To do this, we calculated the similarities

between all products purchased in the main visit. We then calculated the mean similarity between each forgotten product and those purchased in the main shop. For each product removed, we then calculated the percentile of that mean average relative to the distribution of similarities observed between each purchased product.

Results showed that removed products had above average similarity with the purchased products across each knowledge representation. Removed products were, on average, in the 77th percentile ($95\%CI = 0.043$) of episodic similarities, the 62nd percentile ($95\%CI = 0.028$) of semantic similarities and the 79th percentile ($95\%CI = 0.053$) of hierarchical similarities observed between purchased products. This further suggests that products are removed because they are similar to other purchased products (i.e., analogous to confabulations) and not because they are distinct and thus irrelevant to one's goals.

2.11 Search arrival transitions

One concern may be that the design of the website biased shoppers towards retrieving products that were similar to each other. For example, shoppers could be biased towards retrieving hierarchically similar products by the category navigation or by the display of similar products in the search results. We therefore filtered the data so that it only contained transitions between choices before and after the use of a search bar (i.e., search arrivals) and re-ran the analyses reported in the main text. These transitions are perhaps most characteristic of memory-based search. To foreshadow, these analyses reproduce the results presented in the main text, suggesting that they are not an artifact of the website's design.

2.11.1 Permutation tests

Permutation tests were consistent with the results described in the main manuscript. The average trip-wise similarity between consecutively purchased items was significantly higher for the true order of purchases compared to the permuted order for episodic ($Median_{true} = 0.0421, IQR_{true} = 0.0751$ & $Median_{permuted} = 0.0136, IQR_{permuted} = 0.0141$) (Mann-Whitney $U = 379028331647.5$, $p < .0001$, $CLE = 0.7653$), semantic ($Median_{true} = 0.1974, IQR_{true} = 0.1020$ & $Median_{permuted} = 0.0737, IQR_{permuted} = 0.0685$) (Mann-Whitney U = 221679814301.5, $p < .0001$, $CLE = 0.8627$) and hierarchical similarities ($Median_{true} = 0.4556, IQR_{true} = 0.1248$ & $Median_{permuted} = 0.2545, IQR_{permuted} = 0.0483$) (Mann-Whitney U = 150787174769.0, $p < .0001$, $CLE = 0.9077$).

2.11.2 Correlations between representations

As shown in Table S11, there was a small to moderate relationship between each representation of the sequential choices observed in the filtered search-arrival dataset (all significant $p < .0001$). This is consistent with the results shown above.

2.11.3 Timestep regressions

Looking at behaviour over time, the pattern of results was consistent with that described in the main text. The mixed-efects regression converged ($ll = -3345165.74$). Importantly, average response times increased significantly over the duration of the trip ($b_{timestep} = 0.161$), even after accounting for different similarity measures (full regression equation shown in Table S12). Sequential transitions also became increasingly dissimilar over time across representations of episodic ($r_s = -0.2596$, $95\%\ CI\ [-0.2607,\ -0.2585]$, $p <= 0.0001$), semantic ($r_s = -0.0485$, $95\%\ CI\ [-0.0496,\ -0.0474]$, $p <= 0.0001$) and hierarchical knowledge ($r_s = -0.1104$, $95\%\ CI\ [-0.1115,\ -0.1093]$, $p <= 0.0001$).

2.11.4 Representation and IRI regression

We also reproduced the regression analyses of response times using each of the representations as predictors. The mixed-effects linear regression converged ($ll = -3523084.66$) and the full equation is shown in Table S13. As in the main text, the partial regression coefficients for episodic (β = -0.057), semantic (β = -0.034), hierarchical similarity (β = -0.173) were all negatively related to response time. These results are therefore consistent with those shown in the main analyses. As before, hierarchical knowledge explained the most amount of variance in IRIs, even when filtering to memory-based choice transitions that occurred before and after searches.

We also performed feature selection with mixed-effects regressions predicting IRI with each similarity measure. This was to confirm that — as before — IRIs were best explained by a combination of three knowledge representations. As shown in Table S14, fits were best for the model containing all representations.

2.11.5 Retrieval model comparison

We also re-fit the SAM retrieval models to the search only dataset. As shown in Table S15, a model containing multiple representations provided the best fit to the data. As before, hierarchical knowledge received the highest attention weight, further emphasising its importance when retrieving options from memory.

2.11.6 Forgotten and removed item regressions

Finally, we predicted the number of forgotten items and removed products using the attention weights estimated from the SAM model fit to the filtered dataset. The regression predicting the number of forgotten items was significant ($F_{4,98478}$ = 237.2, p $<$ 0.0001, R^2=.010) and — as shown in Table S16 — the coefficients for episodic, semantic and hierarchy attention weights followed the directions reported in the main text. The regression predicting the number of removed items was also significant ($F_{4,98478}$ = 3133, p $<$ 0.0001, R^2=.113) and — as shown in Table S17 — the coefficients followed the directions reported in the main text, with higher

attention to semantic knowledge positively predicting removals and higher attention hierarchical knowledge negatively predicting removals. Note that the coefficient for episodic knowledge was not significant in this regression, although we had no apriori hypotheses about its relationship with removals. This further reinforces the assertion that these results are a product of memory retrieval and not an artifact of the website's design.

As a whole, these results are consistent with the major findings presented in the main text. This supports the claim that these findings reflect memory retrieval processes used by shoppers as they searched for products, given that using the search-bar is perhaps most representative of memory-based search. Despite this, it is important to clarify that other navigation strategies likely reflect memory processes too. For example, shoppers may use their past choice as the basis for determining which category to select next on the drop-down menu. Or choosing a special offer may trigger ideas about related products that are required. This is supported by the fact that results are so similar between the full dataset (reported in the main text) and the search-arrival dataset (reported in this section).

3 Analyses of food fluency data

We further tested the key claims of this article by fitting our retrieval model to data from a controlled experiment that explicitly tested memory retrieval for food (24). In this experiment — originally conducted by Zemla et al. (24) — fifty participants were asked to list as many food items that they could think of within three minutes. Much like searches in a search-bar, each retrieval was typed into a text box (further details can be found in the original article, 24). This experiment therefore makes an excellent testbed for evaluating our model, in that it assesses the role of long-term memory and retrieval in a preferential domain but with a high-degree of experimental control. We evaluated the fit of our model to sequential retrievals from this experiment and — as before — hypothesised that sequential retrievals would be best explained by combining representations of episodic, semantic and hierarchical knowledge.

3.1 Method

3.1.1 Procedure

In the experiment, fifty participants located in the United States were recruited via Amazon Mechanical Turk. Participants completed three separate fluency tasks (animals, tools and foods). Each category was repeated three times and the order of the categories was pseudo-randomised, whilst ensuring that no category was repeated twice. Participants were told not to repeat items within lists but that they could repeat items between lists. Participants had three minutes to complete each list. Each response was typed into a text box one at a time. We restricted our analyses to retrievals from the food category so that we could use the aforementioned embedding spaces, mirroring our analyses of the shopping data.

3.1.2 Data

Data was retrieved from the Github repository (25) associated with the original article (24). For each of the retrievals made in the experiment, we found a matching supermarket product by searching for product descriptions that contained the word and prioritising matches with higher frequency of occurrence in the in-store dataset. A sample of the 40 most-frequently occurring retrievals with their corresponding products is available in Table S18. We were unable to find matching products for a small proportion of the total retrievals (4.64%). Thus, transitions to and from retrievals with no matching products were dropped.

As with the clickstream data, we did not remove repetitions of words, as we believed these repetitions to be informative about one's retrieval process (similar approaches have been taken in other studies of option retrieval, e.g., see 46)).

The final dataset contained 3357 retrievals from 50 participants. Participants retrieved an average of 43.92 unique words ($95\%CI = 4.71$) over all lists and 22.27 unique words ($95\%CI = 1.85$) within each list.

3.1.3 Model

We used the same retrieval model used to explain consumer choices to estimate the probability of each retrieval. As before, we included similarities between the current and all remaining retrievals in the model denominator.

We also used the same fitting procedure as with the consumer choices and compared models containing one, two and three knowledge representations. These were compared with a random baseline, which predicted an equal probability $\frac{1}{n}$ for each choice at each timestep, where n represents the number of retrievals remaining. We therefore report the % BIC improvement over the random baseline for each model.

Due the hierarchical nature of the data (i.e., multiple lists per participant), we estimated parameters for each list separately, for each participant separately (by concatenating each list per participant) and for the first list only. We report separate model comparisons for each of these three estimation procedures.

3.2 Results and discussion

We compared nested models containing different numbers of knowledge representations using three estimating procedures; namely, treating each list separately (results shown in Table S19), each participant separately (collapsing over lists, results shown in Table S20) and to the first list for each participant (results shown in Table S21). As shown in Tables S19, S20 and S21, the pattern of results were consistent between estimation procedures and with those reported in the main text. Namely, models containing multiple knowledge representations provided the best fit to the semantic fluency data (e.g., 9.92% BIC improvement over the random baseline when models were estimated for each list separately). In addition, hierarchical knowledge received the highest attention weight in the multiple representation model, followed by semantic then

episodic knowledge. Hierarchical knowledge also drove the highest improvement in BIC when fit as a single representation. All of these findings are consistent with those reported in the main text.

Thus — across all estimation procedures — we observed a general pattern of results that is strikingly consistent with our major findings. It is reassuring that these results can be recovered from memory retrievals observed in experimental conditions for which there is a high degree of control. This consistency suggests that the online shoppers described in the clickstream dataset depended on similar memory retrieval processes when deciding what to choose next. Moreover, these results support our general claims that past retrievals serve as cues to query multiple sources of long-term knowledge, which combine to determine subsequent retrievals in preferential domains.

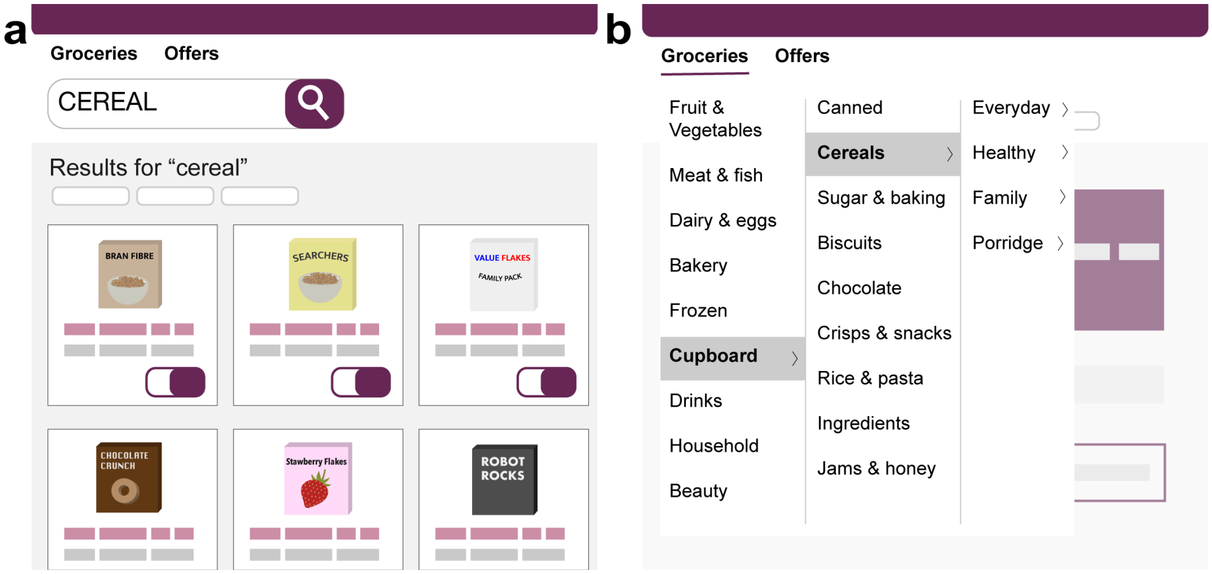

Figure S1: Shoppers could transition between products using different design features of the website. **a.,** Most transitions occurred through use of a search bar, which was located at the top of the page. After entering a keyword, shoppers were presented with a list of relevant products associated with the keyword. Shoppers could add products to their basket from this search results page directly or click on the product to view a dedicated page containing more information (e.g., nutritional data). **b.,** Fewer transitions occurred through use of a category drop-down, which appeared when hovering the mouse over the *Groceries* hyperlink at the top of the page. Three levels of subcategories could be revealed by hovering one’s mouse over the respective department names. In the example pictured, the shopper has hovered their mouse over the “Cupboard” department and then the “Cereals” category. The products in each subcategory were displayed in the same way as the search results.

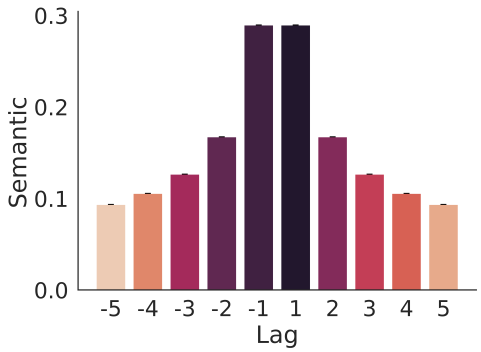

(a) Mean semantic similarity (with 95% confidence intervals) between the current product those purchased most recently is higher compared with products purchased later

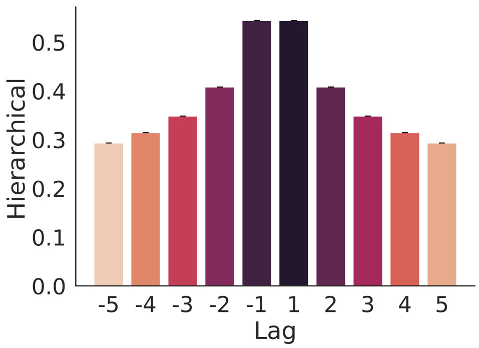

(b) Mean hierarchical similarity (with 95% confidence intervals) between the current product those purchased most recently is higher compared with products purchased later

Figure S2: Lagged average similarity between choices indicates how choices become less similar over time

Table S1: Spearman correlations between each of the similarity measures (95% confidence intervals shown in parentheses)

	Episodic	Semantic	Structured
Episodic		0.585 [0.585, 0.586]	0.526 [0.525, 0.527]
Semantic			0.654 [0.654, 0.655]
Structured			

Table S2: Standardised coefficients, significance values, upper (UB) and lower bounds (LB) for variables included in the mixed-effects linear regression predicting IRI using timestep, transition similarity and transition type

	b	$p \leq$	95% LB	95% UB
Intercept	2.857	0.0001	2.854	2.859
Timestep	0.130	0.0001	0.129	0.130
Episodic similarity	-0.137	0.0001	-0.138	-0.136
Semantic similarity	-0.086	0.0001	-0.087	-0.085
Hierarchical similarity	-0.273	0.0001	-0.274	-0.271
Category arrival	0.157	0.0001	0.136	0.177
Category stay	-0.004	0.680	-0.021	0.014
Offers	0.069	0.0001	0.054	0.084
Search arrival	0.010	0.614	-0.028	0.048
Search stay	-0.135	0.0001	-0.168	-0.103
Suggestions	-0.028	0.0001	-0.035	-0.022
Visit ID	0.162			

Table S3: Standardised coefficients, significance values, upper (UB) and lower bounds (LB) for variables included in the mixed-effects linear regression predicting timestep using episodic similarity and transition type

	b	$p \leq$	95% LB	95% UB
Intercept	5.583	0.0001	5.581	5.585
Episodic similarity	-0.058	0.0001	-0.061	-0.055
Category arrival	0.932	0.0001	0.867	0.997
Category stay	0.758	0.0001	0.702	0.813
Search arrival	1.547	0.0001	1.426	1.668
Search stay	1.346	0.0001	1.242	1.449
Offers	0.448	0.0001	0.400	0.496
Suggestions	0.605	0.0001	0.584	0.626
Visit ID	0.000			

Table S4: Standardised coefficients, significance values, upper (UB) and lower bounds (LB) for variables included in the mixed-effects linear regression predicting timestep using semantic similarity and transition type

	b	$p \leq$	95% LB	95% UB
Intercept	5.583	0.0001	5.581	5.585
Semantic similarity	-0.010	0.0001	-0.013	-0.007
Category arrival	0.948	0.0001	0.883	1.013
Category stay	0.754	0.0001	0.698	0.810
Search arrival	1.577	0.0001	1.456	1.698
Search stay	1.350	0.0001	1.246	1.454
Offers	0.458	0.0001	0.410	0.506
Suggestions	0.609	0.0001	0.587	0.630
Visit ID	0.000			

Table S5: Standardised coefficients, significance values, upper (UB) and lower bounds (LB) for variables included in the mixed-effects linear regression predicting timestep using hierarchical similarity and transition type

	b	$p \leq$	95% LB	95% UB
Intercept	5.583	0.0001	5.581	5.585
Hierarchical similarity	-0.247	0.0001	-0.250	-0.243
Category arrival	0.882	0.0001	0.817	0.947
Category stay	0.784	0.0001	0.728	0.840
Search arrival	1.445	0.0001	1.325	1.566
Search stay	1.403	0.0001	1.299	1.507
Offers	0.422	0.0001	0.374	0.470
Suggestions	0.582	0.0001	0.561	0.603
Visit ID	0.000			

Table S6: Standardised coefficients, significance values, upper (UB) and lower bounds (LB) for variables included in the mixed-effects linear regression model predicting IRI using each similarity measure and confounding variables

	b	$p \leq$	95% LB	95% UB
Intercept	2.834	0.0001	2.831	2.836
Episodic	-0.138	0.0001	-0.139	-0.137
Semantic	-0.083	0.0001	-0.084	-0.082
Hierarchy	-0.278	0.0001	-0.279	-0.277
Category arrival	0.193	0.0001	0.174	0.212
Category stay	0.027	0.0010	0.011	0.044
Search arrival	0.070	0.0001	0.034	0.106
Search stay	-0.081	0.0001	-0.112	-0.050
Offers	0.091	0.0001	0.077	0.105
Suggestions	-0.010	0.0020	-0.017	-0.004
Choices remaining	-0.138	0.0001	-0.139	-0.137
Visit ID	0.161			

Table S7: Nested model comparisons (using AIC and BIC) for linear-mixed effects regressions predicting IRI. Models were chosen using backward elimination.

Variables	AIC	BIC
Episodic, Semantic, Hierarchical and Confounding Variables	12185903.95	12186079.08
Episodic, Hierarchical and Confounding Variables	12204652.31	12204813.97
Hierarchical and Confounding Variables	12367250.26	12367398.45

Table S8: Spearman correlations between each of the attention weights

	Episodic	Semantic	Hierarchy
Episodic	-	0.0216 [0.0159, 0.0273]	-0.2733 [-0.2786, -0.268]
Semantic	-	-	-0.2627 [-0.268, -0.2574]
Hierarchy	-	-	-

Table S9: Standardised coefficients, significance values, upper (UB) and lower bounds (LB) for variables included in the regression model predicting number of forgotten items using the model attention weights, total number of choices and proportion of each transition type

	b	$p \leq$	95% LB	95% UB
Intercept	0.3044	0.0001	0.299	0.310
Episodic	-0.0534	0.0001	-0.059	-0.048
Semantic	0.0150	0.0001	0.009	0.021
Hierarchy	-0.0407	0.0001	-0.046	-0.035
Category arrival	-0.0065	0.1660	-0.016	0.003
Category stay	-0.0187	0.0001	-0.028	-0.010
Offers	0.0454	0.0001	0.039	0.052
Search arrival	-0.0406	0.0001	-0.052	-0.029
Total choices	-0.0414	0.0001	-0.047	-0.036

Table S10: Standardised coefficients, significance values, upper (UB) and lower bounds (LB) for variables included in the regression model predicting number of products removed using the model attention weights, total number of choices and proportion of each transition type

	b	$p \leq$	95% LB	95% UB
Intercept	3.3836	0.0001	3.347	3.420
Episodic	-0.3585	0.0001	-0.398	-0.319
Semantic	0.0506	0.0090	0.012	0.089
Hierarchy	-0.8702	0.0001	-0.909	-0.831
Category arrival	0.2135	0.0001	0.151	0.277
Category stay	-0.2850	0.0001	-0.345	-0.225
Offers	0.3090	0.0001	0.264	0.354
Search arrival	-0.0507	0.1990	-0.128	0.027
Total choices	2.3466	0.0001	2.309	2.385

Table S11: Spearman correlations between each of the similarity measures in the search-arrival dataset ($N = 3086716$

	Episodic	Semantic	Hierarchy
Episodic	-	0.3254 [0.3244, 0.3264]	0.2192 [0.2181, 0.2203]
Semantic	-	-	0.3217 [0.3207, 0.3227]
Hierarchy	-	-	-

Table S12: Standardised coefficients, significance values, upper (UB) and lower bounds (LB) for variables included in the mixed-effects linear regression predicting IRI using timestep, fit to the search-arrival dataset ($N = 2959878$)

	b	$p \leq$	95% LB	95% UB
Timestep	0.161	0.0001	0.160	0.162
Episodic similarity	-0.050	0.0001	-0.051	-0.049
Semantic similarity	-0.036	0.0001	-0.036	-0.035
Hierarchical similarity	-0.169	0.0001	-0.170	-0.168
Group Var	0.228			

Table S13: Standardised coefficients, significance values, upper (UB) and lower bounds (LB) for variables included in the mixed-effects linear regression model predicting IRI using each similarity measure, fit to the search-arrival dataset

	b	$p \leq$	95% LB	95% UB
Intercept	3.092	0.0001	3.089	3.095
Episodic	-0.057	0.0001	-0.058	-0.056
Semantic	-0.034	0.0001	-0.035	-0.033
Hierarchy	-0.173	0.0001	-0.174	-0.172
Choices remaining	-0.172	0.0001	-0.173	-0.171
Visit ID	0.238			

Table S14: Nested model comparisons (using AIC and BIC) for regressions predicting IRI using each similarity measure, fit to the search-arrival dataset. Models were chosen using backward elimination.

Variables	AIC	BIC
Episodic, Semantic, Hierarchical and Choices remaining	7046183.33	7046273.93
Episodic, Hierarchical and Choices remaining	7051248.70	7051326.35
Hierarchical and Choices remaining	7071024.10	7071088.81

Table S15: The % BIC improvement over the random baseline and the mean attention weights (with 95% confidence intervals) for each of the candidate retrieval models, fit to the search-arrival dataset. Results show that including representations of all knowledge formats provides the best fit to the data (shown in bold)

	Δ BIC (%)	Episodic	Semantic	Hierarchy
Episodic	8.28	0.309 (0.001)		
Semantic	4.37		0.093 (0.001)	
Hierarchy	27.80			2.398 (0.021)
Episodic & Semantic	11.20	0.276 (0.001)	0.068 (0.001)	
Semantic & Hierarchy	30.47		0.057 (0.001)	2.306 (0.024)
Episodic & Hierarchy	32.63	0.187 (0.001)		2.193 (0.022)
Multiple	**34.59**	0.172 (0.001)	0.046 (0.001)	2.144 (0.025)

Table S16: Standardised coefficients, significance values, upper (UB) and lower bounds (LB) for variables included in a multiple linear regression predicting the number of forgotten items, fit to the search-arrival dataset

	b	$p \leq$	95% LB	95% UB
Intercept	0.2904	0.0001	0.285	0.296
Episodic	-0.0467	0.0001	-0.052	-0.041
Semantic	0.0065	0.0190	0.001	0.012
Hierarchy	-0.0565	0.0001	-0.062	-0.051
Choices remaining	-0.0486	0.0001	-0.054	-0.043

Table S17: Standardised coefficients, significance values, upper (UB) and lower bounds (LB) for variables included in a multiple linear regression predicting the number of removed items, fit to the search-arrival dataset

	b	$p \leq$	95% LB	95% UB
Intercept	3.4484	0.0001	3.407	3.490
Episodic	-0.0048	0.8230	-0.047	0.037
Semantic	0.2305	0.0001	0.189	0.272
Hierarchy	-0.9995	0.0001	-1.042	-0.957
Total choices	2.2511	0.0001	2.209	2.293

Table S18: Matching retailer products for the 40 most frequently occurring retrievals reported by (24). Note that brand names have been redacted.

item	Product	No. occurrences
apple	GALA APPLE MINIMUM 5 PACK	101
pizza	MARGHERITA PIZZA 245G	95
chicken	FREE RANGE WHOLE CHICKEN 1KG-2.3KG	90
banana	BANANAS LOOSE	80
bread	SLICED WHITE BREAD 800G	76
cheese	MATURE CHEDDAR CHEESE 350 G	76
carrot	CARROTS LOOSE	74
orange	PURE ORANGE JUICE SMOOTH 1 LTR	70
steak	ABERDEEN ANGUS STEAK MINCE 500G	64
icecream	SOFT SCOOPVANILLA 2 LITRES	63
grape	SEEDLESS GRAPES 500G	62
potato	MARIS PIPER POTATOES 2.5KG	61
tomato	SALAD TOMATOES 6 PACK	61
hamburger	4 BRITISH BEEF STEAK BURGERS 454G	61
spaghetti	SHORT SPAGHETTI PASTA 500G	58
cake	CHOCOLATE CAKE ROLL 10 PACK	58
rice	M/WAVE BASMATI RICE 250G	58
broccoli	BROCCOLI LOOSE	57
lettuce	ICEBERG LETTUCE EACH	56
corn	CORN ON THE COB TWINPACK	52
strawberry	STRAWBERRIES 400G	52
onion	BROWN ONIONS LOOSE	51
egg	MEDIUM FREE RANGE EGGS 6 PACK	49
turkey	BRITISH ROAST TURKEY SLICES 125 G	49
frenchfry	CRISPY FRENCH FRIES 900G	46
taco	CRNCHY TACO SHELLS X12 156G	44
beans	BEANS IN TOMTO SAUCE 415G	43
hotdog	CLASSIC FRANKFURTER HOT DOGS 10 PK 350G	43
pasta	FUSILLI PASTA TWISTS 1KG	43
spinach	ORGANIC SPINACH 200G	42
cereal	VARIETY PACK CEREAL 8 PACK	42
ham	HAM SLICES 125 G	42
soup	CREAM OF TOMATO SOUP 400G	41
yogurt	GREEK STYLE YOGHURT 500G	41
pear	CONFERENCE PEARS PACK 610G	41
bacon	UNSMOKED BACK BACON RASHERS 300G	40
sushi	SUSHI NORI 11G	40
beef	LEAN BEEF STEAK MINCE 5% FAT 250G	39
pie	TEAK & ALE PUFFPASTRY PIE 500G	39
pineapple	PINEAPPLE LOOSE	39

Table S19: The % BIC improvement over the random baseline and the mean attention weights (with 95% confidence intervals) for each of the candidate retrieval models, *fit to each retrieval list separately*. Results show that including representations of all knowledge formats provides the best fit to the data (shown in bold)

	Δ BIC (%)	Episodic	Semantic	Hierarchy
Semantic	1.496		0.089 (0.073)	
Episodic	2.176	0.207 (0.06)		
Hierarchy	7.818			2.071 (0.874)
Episodic & Semantic	3.247	0.922 (1.087)	1.702 (2.62)	
Semantic & Hierarchy	8.750		0.055 (0.041)	1.894 (0.724)
Episodic & Hierarchy	9.153	0.223 (0.149)		2.347 (1.461)
Multiple	**9.917**	0.669 (0.753)	0.282 (0.474)	3.777 (3.082)

Table S20: The % BIC improvement over the random baseline and the mean attention weights (with 95% confidence intervals) for each of the candidate retrieval models, *fit to each participant and collapsing over multiple lists*. Results show that including representations of all knowledge formats provides the best fit to the data (shown in bold)

	Δ BIC (%)	Episodic	Semantic	Hierarchy
Semantic	0.910		0.031 (0.007)	
Episodic	1.120	0.104 (0.033)		
Hierarchy	6.879			1.212 (0.156)
Episodic & Semantic	1.798	0.092 (0.032)	0.026 (0.007)	
Episodic & Hierarchy	7.359	0.067 (0.028)		1.166 (0.156)
Semantic & Hierarchy	7.364		0.021 (0.007)	1.166 (0.159)
Multiple	**7.760**	0.06 (0.028)	0.018 (0.007)	1.129 (0.158)

Table S21: The % BIC improvement over the random baseline and the mean attention weights (with 95% confidence intervals) for each of the candidate retrieval models, *fit to the first retrieval list from each participant*. Results show that including representations of all knowledge formats provides the best fit to the data (shown in bold)

	Δ BIC (%)	Episodic	Semantic	Hierarchy
Semantic	0.722		0.028 (0.01)	
Episodic	1.194	0.133 (0.05)		
Hierarchy	5.998			1.307 (0.57)
Episodic & Semantic	1.746	0.135 (0.058)	0.025 (0.01)	
Semantic & Hierarchy	6.500		0.022 (0.01)	1.279 (0.577)
Episodic & Hierarchy	6.693	0.095 (0.046)		1.254 (0.568)
Multiple	**7.175**	0.174 (0.191)	0.032 (0.029)	1.323 (0.743)

REFERENCES AND NOTES

1. P. W. Glimcher, A. Rustichini, Neuroeconomics: The consilience of brain and decision. *Science* **306**, 447–452 (2004).

2. J. R. Busemeyer, J. Rieskamp, *Handbook of Choice Modelling* (Edward Elgar Publishing, 2014).

3. A. Rangel, C. Camerer, P. R. Montague, A framework for studying the neurobiology of value-based decision making. *Nat. Rev. Neurosci.* **9**, 545–556 (2008).

4. L. Keller, J. Ho, Decision problem structuring: Generating options. *IEEE Trans. Syst. Man, Cybernetics* **18**, 715–728 (1988).

5. A. Kalis, S. Kaiser, A. Mojzisch, Why we should talk about option generation in decision-making research. *Front. Psychol.* **4**, 555 (2013).

6. Z. Zhang, A. Richards, M. I. Barrasa, S. H. Hughes, R. A. Young, R. Jaenisch, Reverse-transcribed SARS-CoV-2 RNA can integrate into the genome of cultured human cells and can be expressed in patient-derived tissues. *Proc. Natl. Acad. Sci.* **118**, e2105968118 (2021).

7. S. Bhatia, Semantic processes in preferential decision making. *J. Exp. Psychol. Hum. Learn.* **45**, 627–640 (2019).

8. W. A. Bousfield, C. H. W. Sedgewick, An analysis of sequences of restricted associative responses. *J. Gen. Psychol.* **30**, 149–165 (1944).

9. A. K. Troyer, M. Moscovitch, G. Winocur, Clustering and switching as two components of verbal fluency: Evidence from younger and older healthy adults. *Neuropsychology* **11**, 138–146 (1997).

10. P. J. Gruenewald, G. R. Lockhead, The free recall of category examples. *J. Exp. Psychol. Hum. Learn.* **6**, 225–240 (1980).

11. T. T. Hills, M. N. Jones, P. M. Todd, Optimal foraging in semantic memory. *Psychol. Rev.* **119**, 431–440 (2012).

12. J. T. Abbott, J. L. Austerweil, T. L. Griffiths, Random walks on semantic networks can resemble optimal foraging. *Psychol. Rev.* **122**, 558–569 (2015).

13. M. Proust, *In Search of Lost Time* (Modern Library, 1913).

14. H. Ebbinghaus, *Memory: A Contribution to Experimental Psychology* (Teachers College Press, 1913).

15. M. J. Kahana, Computational models of memory search. *Annu. Rev. Psychol.* **71**, 107–138 (2020).

16. M. N. Jones, D. J. K. Mewhort, Representing word meaning and order information in a composite holographic lexicon. *Psychol. Rev.* **114**, 1–37 (2007).

17. A. M. Collins, M. R. Quillian, Retrieval time from semantic memory. *J. Verbal Learn. Verbal Behav.* **8**, 240–247 (1969).

18. J. G. Johnson, M. Raab, Take the first: Option-generation and resulting choices. *Organ. Behav. Hum. Decis. Process.* **91**, 215–229 (2003).

19. G. Klein, S. Wolf, L. Militello, C. Zsambok, Characteristics of skilled option generation in chess. *Organ. Behav. Hum. Decis. Process.* **62**, 63–69 (1995).

20. A. N. Hornsby, T. Evans, P. S. Riefer, R. Prior, B. C. Love, Conceptual organization is revealed by consumer activity patterns. *Comput. Brain Behav.* **3**, 162–173 (2020).

21. T. Mikolov, I. Sutskever, K. Chen, G. Corrado, J. Dean, Distributed representations of words and phrases and their compositionality, in *Proceedings of the 26th International Conference on Neural Information Processing Systems - Volume 2*, NIPS'13 (Curran Associates Inc., 2013), pp. 3111–3119.

22. J. G. W. Raaijmakers, R. M. Shiffrin, *Psychology of Learning and Motivation*, G. H. Bower, Ed. (Academic Press, 1980), vol. 14, pp. 207–262.

23. M. L. Mack, A. R. Preston, B. C. Love, Ventromedial prefrontal cortex compression during concept learning. *Nat. Commun.* **11**, 46 (2020).

24. J. C. Zemla, K. Cao, K. D. Mueller, J. L. Austerweil, SNAFU: The semantic network and fluency utility. *Behav. Res. Methods* **52**, 1681–1699 (2020).

25. J. C. Zemla, K. Cao, K. D. Mueller, J. L. Austerweil, SNAFU: The Semantic Network and Fluency Utility, Dataset (2020); https://github.com/AusterweilLab/snafu-py [accessed 13 October 2021]

26. R. M. Shiffrin, Forgetting: Trace erosion or retrieval failure? *Science* **168**, 1601–1603 (1970).

27. M. C. Anderson, R. A. Bjork, E. L. Bjork, Remembering can cause forgetting: Retrieval dynamics in long-term memory. *J. Exp. Psychol. Learn* **20**, 1063 (1994).

28. J. R. Bettman, Memory factors in consumer choice: A review. *J. Market.* **43**, 37–53 (1979).

29. J. R. Anderson, A spreading activation theory of memory. *J. Verbal Learn. Verbal Behav.* **22**, 261–295 (1983).

30. A. M. Collins, E. F. Loftus, A spreading-activation theory of semantic processing. *Psychol. Rev.* **82**, 407–428 (1975).

31. J. Deese, On the prediction of occurrence of particular verbal intrusions in immediate recall. *J. Exp. Psychol.* **58**, 17–22 (1959).

32. H. L. Roediger, K. B. McDermott, Creating false memories: Remembering words not presented in lists. *J. Exp. Psyhol. Learn* **21**, 803 (1995).

33. J. Avery, M. N. Jones, *CogSci* (2018).

34. S. Kaiser, J. J. Simon, A. Kalis, S. Schweizer, P. N. Tobler, A. Mojzisch, The cognitive and neural basis of option generation and subsequent choice. *Cogn. Affect. Behav. Neurosci.* **13**, 814–829 (2013).

35. D. Mirman, J.-F. Landrigan, A. E. Britt, Taxonomic and thematic semantic systems. *Psychol. Bull.* **143**, 499–520 (2017).

36. D. J. Levy, P. W. Glimcher, Comparing apples and oranges: Using reward-specific and reward-general subjective value representation in the brain. *J. Neurosci.* **31**, 14693–14707 (2011).

37. A. N. Hornsby, B. C. Love, How decisions and the desire for coherency shape subjective preferences over time. *Cognition* **200**, 104244 (2020).

38. E. Tulving, How many memory systems are there?*Am. Psychol.* **40**, 385–398 (1985).

39. T. L. Griffiths, Manifesto for a new (computational) cognitive revolution. *Cognition* **135**, 21–23 (2015).

40. K. D. Mueller, R. L. Koscik, A. LaRue, L. R. Clark, B. Hermann, S. C. Johnson, M. A. Sager, Verbal fluency and early memory decline: Results from the wisconsin registry for Alzheimer's prevention. *Arch. Clin. Neuropsychol.* **30**, 448–457 (2015).

41. J. Rasmussen, H. Langerman, Alzheimer's disease—Why we need early diagnosis. *Degener. Neurol. Neuromuscul. Dis.* **Volume 9**, 123–130 (2019).

42. R. L. Goldstone, G. Lupyan, Discovering psychological principles by mining naturally occurring data sets. *Topics Cognitive Sci.* **8**, 548–568 (2016).

43. M. N. Jones, J. Willits, S. Dennis, *Models of Semantic Memory* (Oxford Univ. Press, 2015).

44. R. Agrawal, T. Imielinski, A. Swami, Mining association rules between sets of items in large databases, in *Proceedings of the 1993 ACM SIGMOD International Conference on Management of Data* (ACM, 1993), pp. 207–216.

45. K. L. Stachenfeld, M. M. Botvinick, S. J. Gershman, The hippocampus as a predictive map. *Nat. Neurosci.* **20**, 1643–1653 (2017).

46. A. Aka, S. Bhatia, What I like is what I remember: Memory modulation and preferential choice. *J. Exp. Psychol. General* **150**, 2175–2184 (2021).
